# Facile synthesis of MOF-derived N doped ZnO/C nanoparticles and its adsorption activity toward dye removal

**DOI:** 10.1186/s13065-023-01038-6

**Published:** 2023-09-26

**Authors:** Khadiga Mohamed Abas, Sherief A. Al Kiey

**Affiliations:** 1 Laboratory of Surface Chemistry and Catalysis, National Research Centre, 33 El-Bohouth St., Giza, 12622 Egypt; 2 Electrochemistry and Corrosion Laboratory, Physical Chemistry Department, National Research Centre, Dokki, Cairo, 12622 Egypt; 3 Material Engineering Lab, Central laboratories Network, National Research Centre, Dokki, Cairo, 12622 Egypt

**Keywords:** MOFs, ZIF-8, Carbonization, Adsorption

## Abstract

Metal–organic framework (MOF)-derived materials have gained an increasing interest and showed potential adsorption features in numerous applications. Significant attempts have been performed to boost the structure, functionality, surface area and porosity in addition to adsorption performance of MOF-derived carbon nanoparticles. Here, nitrogen-doped ZnO/carbon nanoparticles were synthesized by directly pyrolysis of Zn based metal organic framework (ZIF-8) in a nitrogen atmosphere at two different temperatures (600 and 800 °C), followed by chemical impregnation with ZnCl_2_ solution with ratio (10:1) *wt/wt*, and thermal activation at 500 °C for 1 h. SEM, TEM, XPS, nitrogen adsorption–desorption method, and TGA characterization techniques were employed to investigate the morphology and structure characteristics. Then, thorough analysis of N doped ZnO/C-(600 and 800), adsorption capacity to remove Remazol brilliant blue reactive (RBBR) dye from aqueous phase was conducted. At room temperature, the porous N doped ZnO/C with high surface area attained a maximum adsorption capacity about 49.3 mg/g and demonstrated a strong adsorption capacity toward RBBR dye. The insights of kinetic, thermodynamic and adsorption isotherm studies of the as-demonstrated samples open up more discussion for MOFs-derived carbon adsorbents for wastewater treatment.

## Introduction

Metal–organic frameworks (MOFs) are porous coordination polymers with regular pores generated by the coordination of metal ions or metal clusters with organic bridging molecules (ligands) [1, 2]. MOF materials are of specific importance because of their facile tunability of pore size and large surface area [3]. They are extensively used in the fields of catalysis [4, 5], gas and vapor adsorption [6], Corrosion inhibition [7], sensing [8], and energy applications [9, 10]. A moderate adsorbing capacity was attained with numerous MOF materials, including UiO-66 [11], and MIL-53 [12]. The MOF/metal oxide composites are typically made either by self-assembling MOFs onto the surface of metal oxide nanoparticles, rods, or wires that have already been synthesized or by a metal oxide forming in or on previously formed MOFs.

(MOFs) have recently been demonstrated to be the best sacrificial templates for fabricating a variety of carbon-based nanomaterials, including porous carbons, heteroatom-doped porous carbons, and decorated porous carbons with metal or metal oxides, via thermal decomposition under controlled conditions [13]. The carbon-based nanomaterials made by this innovative MOF-templated approach have numerous benefits over the hard- or soft-template methods, including high specific surface area, adjustable porosity, and ease of functionalization with additional heteroatoms or metal/metal oxides. Accordingly, by pyrolysis MOFs at an appropriate temperature and inert environment, the metal ions can be converted into metal nanoparticles (NPs), metal oxide nanostructures, or both relying on the reduction potential of the metal atoms existing in the MOFs [14]. As a metal–organic framework (MOF) containing nitrogen, the zeolitic imidazolate framework-8 (ZIF-8), is created by the coordination of Zn^2+^ and 2-methylimidazole [15]. Variable porosity, a substantial specific surface area, a stable structure, and elasticity are all features of the ZIF-8-derived carbon that are widely desired after in energy storage applications [10]. Several dyes display high and quick adsorption onto the surface of ZIF-8-derived materials due to the bonding between colors and zinc oxide (ZnO) [16]. It is essential to note that MOF-derived carbon possesses several unique and tunable pore structures such as high surface area, controllable pore size, and surface functionality. The MOF-derived carbon possesses enhanced textural properties, which make it an excellent material for adsorption-based applications. These properties make MOF-derived carbon more efficient in adsorption-based applications, such as dye removal from wastewater. Although MOF-based materials are not always economically feasible to prepare, the carbonization of MOF precursors offers a simple and cost-effective approach to synthesize highly porous carbon materials [17].

The Excessive dye discharge in wastewater is a hazard for the environment and can destroy the ecosystem by reducing the rate of photosynthesis [18], light penetration and toxicity of heavy metals found in pigments [19]. Dyes are complex aromatic molecular structures with a synthetic origin. To color manufactured product, synthetic dyes are extensively employed in sectors including leather, paper, textiles and plastics. The pulp and textile industries use a large amount of water in their manufacturing processes, which results in a significant amount of wastewater. These industries effluents are also recognized by their low biochemical oxygen demand (BOD) and oversaturated colors content. The most extensively used dyes are reactive dyes because of their vivid colors, outstanding colorfastness, simplicity of use, minimal energy demand, and high solubility in water. The examples of reactive dyes include remazol brilliant blue R (RBBR), remazol brilliant violet 5R (RBV) and remazol black (RB). Water wasted with reactive dyes are extremely carcinogenic and hazardous to organisms when discharged into receiving streams as it decreases photosynthetic activity. Due to these dye’s resilience to biodegradation, oxidants, heat, and light, the removal is one of the key challenges in treating this kind of effluents and they cannot be removed using traditional treatment approaches [20, 21]. Conventional treatments including membrane processes, biological, coagulation, and electrochemical methods are typically inadequate for completely removing color [22]. Numerous different techniques, including adsorption on organic and inorganic substrates, photocatalysis, chemical oxidation, microbiological or enzymatic degradation, have been devised [23]. Since retention onto solid surfaces is an efficient and affordable physical approach that might enables a full de-colorization of the wastewater and their potential re-use, it has been recognized as a treatment of choice for dye removal [24]. The most widely used adsorbents for separating the pollutants are activated carbon, natural fibers, carbon nanotubes [25], carbon fibers, and material-based polymer composites [26]. ZIF-8-derived carbon is very promising to be employed as high-efficient adsorbent to remove specific types of dyes from water because of the evenly distributed zinc ions inside the pores and onto the high surface area (> 1000 m^3^/g).

The following study was carried out with the aim to prepare MOFs-derived a novel carbon nanomaterials called N doped ZnO/C in order to preparing a detailed discussion of the mechanism underlying porous N doped ZnO/C in addition to the high adsorption selectivity for RBBR over other dyes is provided involving adsorption kinetics, thermodynamic and isotherm studies.

## Materials and methods

### Chemicals

Chemicals including (zinc nitrate hexahydrate, 2-methylimidazole, ammonium hydroxide solution, zinc chloride ZnCl_2_ and s were purchased from Alfa Aeser and utilized as received, with no further purification. Remazol brilliant blue reactive dye (RBBR) was gained from (Sigma-Aldrich).

### Sample preparation

ZIF-8 was synthesized in accordance with earlier research [10]. Typically, 2-methylimidazole (3.28 g) and zinc nitrate hexahydrate (1.485 g) were individually dissolved in pure methanol (50 mL). The zinc nitrate hexahydrate solution was then immediately mixed with 2-methylimidazole solution. To finish the crystallization process, the resulting combined solution was agitated for 2 h at room temperature. After obtaining the precipitate through centrifugation, it was thoroughly cleaned with 100% methanol and nominated as ZIF-8. ZIF-8 was directly carbonized for 3 h at 600 and 800 °C under N_2_ gas to produce N doped ZnO/C-600 and N doped ZnO/C-800, respectively.

### Activation of As-synthesized N doped ZnO/C specimens

Chemical activation with zinc chloride was used by means of an impregnation procedure to accomplish the activation process. The experimental set-up for the preparation of activated N doped ZnO/C samples by impregnating treatment was reliant on the proportion of activating agent/N doped ZnO/C samples (1: 10 (wt/wt)) at room temperature. The mixture was first maintained under atmospheric pressure at 100 °C overnight in order to saturate as-synthesized samples with zinc chloride solution. Then, the mixture was thermally activated for 1 h inside a muffle furnace at 500 °C incrementally with an annealing rate 3 °C/min (Cherik and Louhab 2017). Eventually, the activated samples were rinsed in a diluted HCl (0.1 N) with liquid: solid ratio (10 mL/1 g), then retained for 1 h and washed repeatedly with hot distilled water till neutral pH and chloride ions were detached (Nath et al. 2013). Once the activating agent was eliminated, the activated samples were dried at 120 °C for 12 h.

### Characterization

Scanning electron microscopy (SEM) was used on a (QUANTA FEG 250) to look at the morphologies of the materials. The X-ray patterns were acquired using a Bruker diffractometer with a CuK radiation source (λ = 1.540598), and a diffraction angle ranging from 10 to 80°. Using a Quantachrome, model NOVA 1200e, the BET surface area (S_BET_), total pore volume (V_T_), micropore volume (V_m_), average pore diameter (D_P_), and pore size distribution were calculated using N_2_ adsorption and desorption isotherms at 77 K. The S_BET_ value was calculated by fitting the Brunauer–Emmett–Teller (BET) equation linearly across the relative pressure (p/p_0_) range of 0.053 to 0.183. At p/p_0_ = 0.99, the V_T_ represents the highest quantity of N_2_ adsorbed, and V was calculated using the Boer technique. The thermograms (TG), produced by a thermogravimetric analyzer from Themsys one, (France) with a N_2_ flow rate of 100 mL/min and a heating rate of 10 °C/min from room temperature to 850 °C, were used to evaluate the thermal behavior of the materials.

### Dye characteristics

Various concentrations from Remazol Brilliant Blue Reactive dye (RBBR) were prepared in a range between 20 and 200 mg/L in a distilled water solvent. The features and chemical structure of the dye are listed in Table 1 [27].

**Table 1:** Characteristics of RBBR dye

Molecular structure	
Molecular formula	C_22_H_16_N_2_Na_2_O_11_S_3_
Molecular weight	626.54
λ_max_(nm)	595

### Adsorption characteristics

The destination of the current study is to investigate the exploitation of N-doped ZnO/C specimens to remediate aqueous solutions contaminated with RBBR dye. Consequently, stock solutions with diverse concentrations were prepared by dissolution (20–200) mg of RBBR dye in 1 L of distilled water. Thereafter, 10 mg of adsorbents at neutral pH (6.5) was administrated with 10 mL RBBR dye without any external adjustment. To establish equilibrium, every sample was held in a rotary shaker at 220 r.p.m for 48 h at room temperature. Preliminary tests revealed that a time of 48 h was adequate to attain equilibrium conditions. The UV–visible absorption spectra of the supernatant solution were examined utilizing a Shimadzu UV–visible spectrophotometer (Type UV-2401PC) in a 1 cm quartz cuvette to track the characteristic absorption peaks of RBBR dye at a wavelength of 590 nm. The amount of adsorption capacity at equilibrium [q_e_, (mg/g)] was calculated using the following equation:

1 $$q_{e} = \frac{{V \left( {C_{o} - C_{e} } \right)}}{m}$$

where C_o_ and C_e_ (mg/L) are the initial and equilibrium dye concentrations, V (L) is the volume of the equilibrium solution, and m (g) is the mass of the adsorbent.

The effect of variables that influence the adsorption capacity of RBBR dye was then explored. These variables comprise the initial adsorbate concentration (20–200 mg/L), the impact of the contact time (1/2 h-48h), and the influence of temperature (35, 45 and 55° C). Subsequently, the adsorption isotherms, kinetic, and thermodynamic studies were explored.

## Results and discussion

### Characterization of N doped ZnO/C

**Fig. 1:**
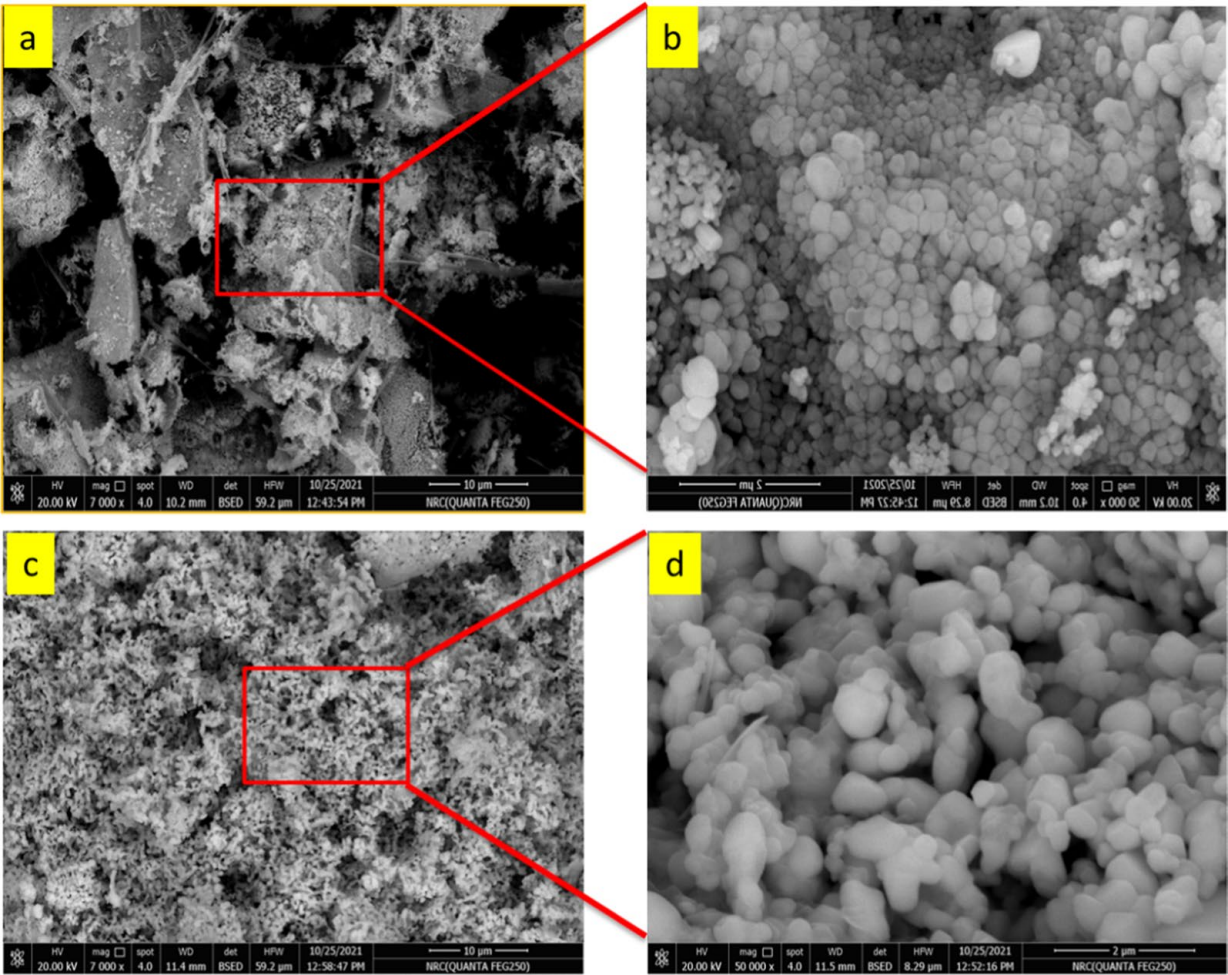
SEM images of N doped porous ZnO/C nanoparticles carbonized at **a** 600 °C (**b** higher magnification), and **c** 800 °C (**d** higher magnification)

**Fig. 2:**
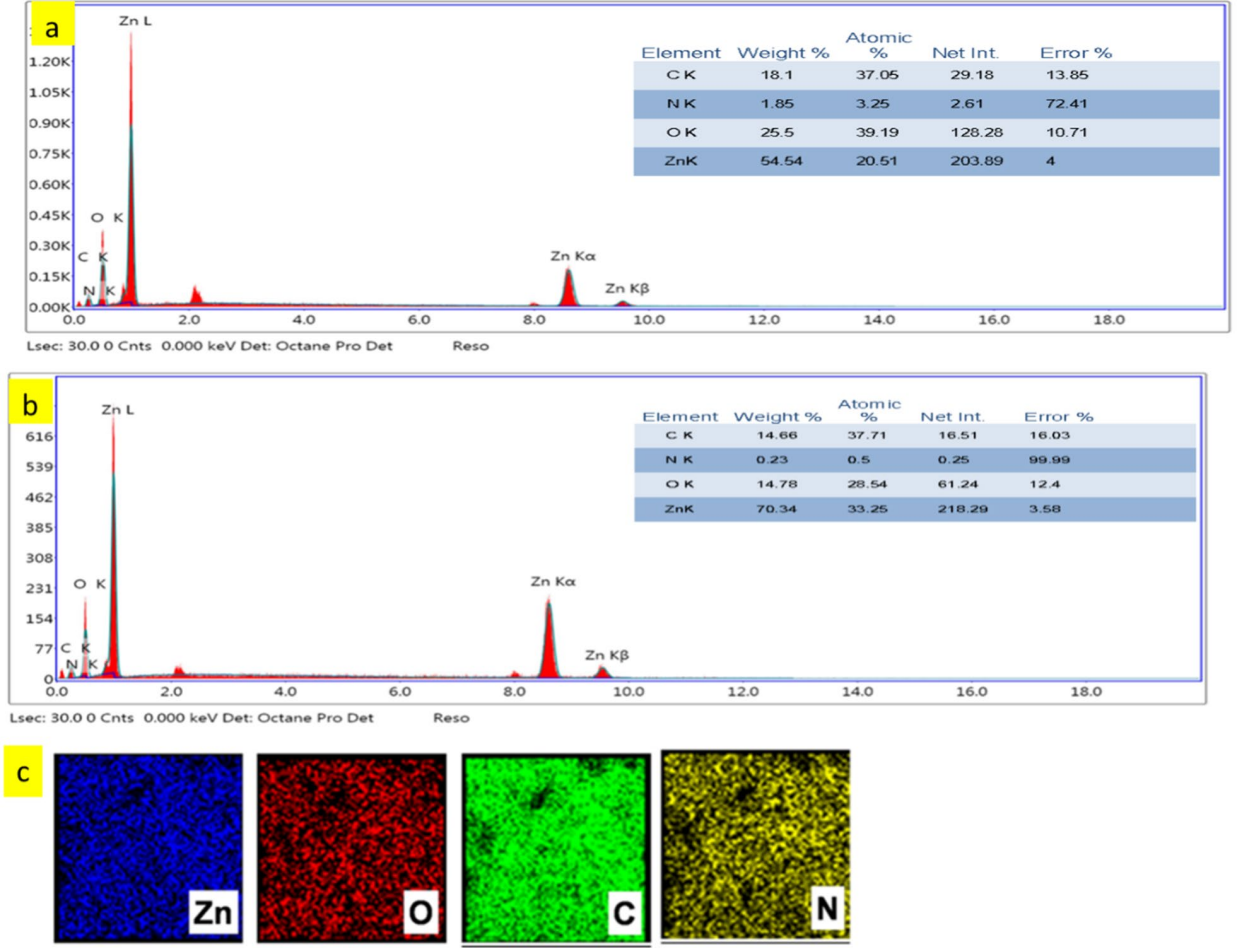
EDX of **a** N ZnO/C-600, **b** N ZnO/C-800 nanoparticles, and **c** elemental mapping of N ZnO/C-600

The morphology of N doped ZnO/C-600 and N doped ZnO/C-800 obtained by carbonizing pure ZIF-8 is shown by the SEM images in Fig. 1. Figure 1 displays SEM images of N doped ZnO/C-600 and N doped ZnO/C-800 at various magnifications (a–d). N doped ZnO/C-600 and N doped ZnO/C 800-have irregular granular features in the SEM images, with a wide dispersion of particles ranging in size around 85 nm and 220 nm, respectively. A characteristic porous dodecahedron morphology is observed. The EDX was also used to evaluate the elemental composition of the as-synthesized materials. The results of an EDS investigation into the elemental compositions of N doped ZnO/C are displayed in Fig. 2. It could be determined that the N doped ZnO/C contained four evenly distributed elements; C, N, O, and Zn. The elemental mapping shows the elemental distribution and the presence of the Zn, O, C and N as shown in Fig. 2c

**Fig. 3:**
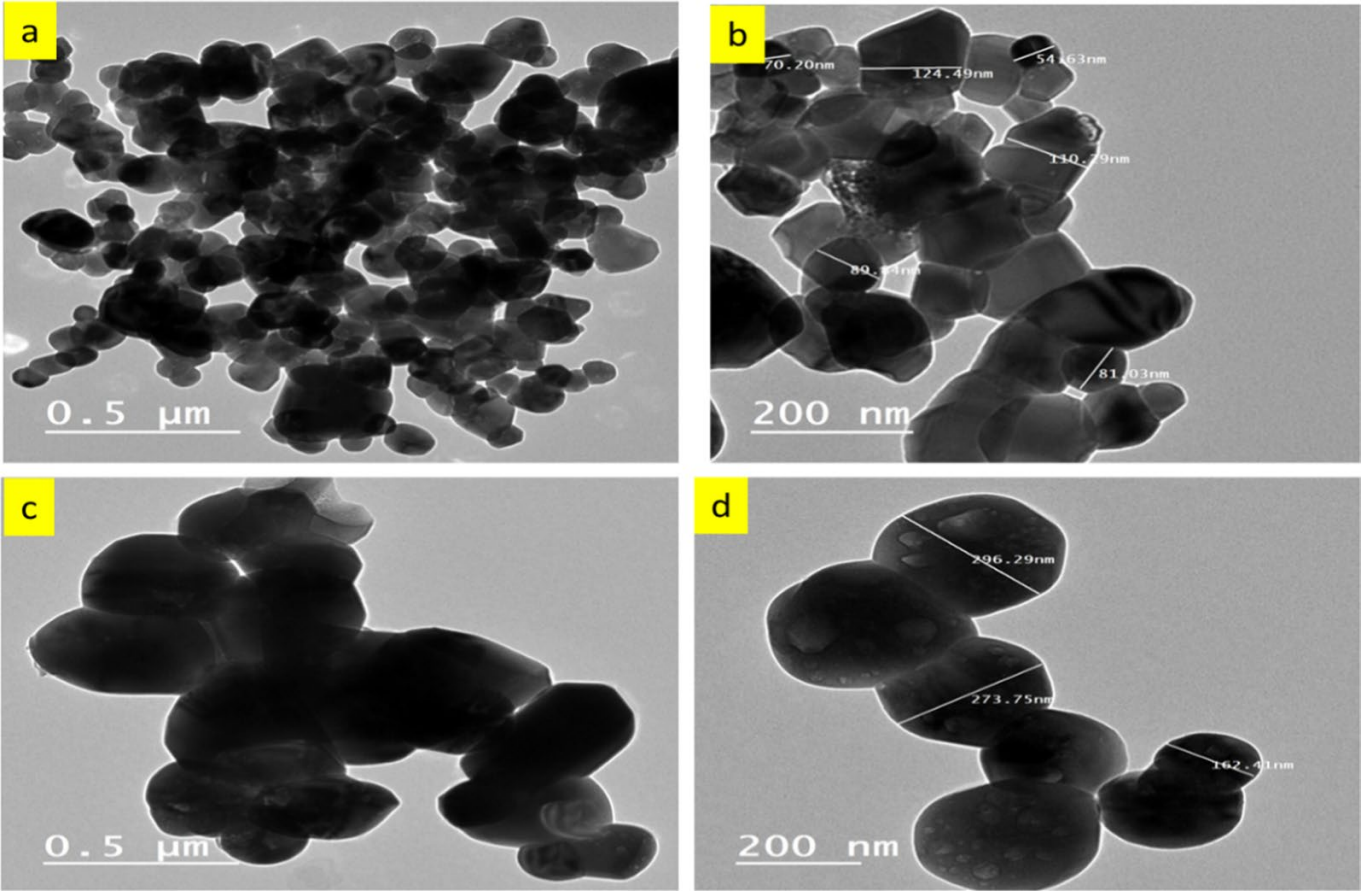
Transmission electron micrographs (TEM) of **a**, **b** N doped ZnO/ C-600 and **c** **d** N doped ZnO/ C-800

The TEM was utilized to characterize N-doped ZnO/C-600 and N-doped ZnO/C-800 obtained from pyrolysis of ZIF-8 in order to better investigation for the fine structural properties of the material. The typical TEM images of the samples were displayed with various magnifications as illustrated in Fig. 3. It demonstrates that the ZnO particles from ZIF-8 are consisted of a lot of tiny nano hexagonal particles, which is in agreement with the SEM. Presumably, the C is the source of the image’s dark spread.

**Fig. 4:**
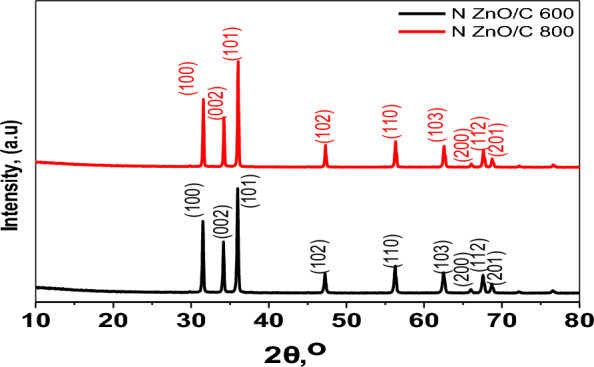
XRD diffraction pattern of N doped ZnO/C samples at different carbonization temperatures

The diffraction characteristics of the nanocomposites, illustrated by the analysis of XRD in Fig. 4 revealed their crystalline features. Based on JCPDS file no. 04-015-4060, the ZnO/C diffraction patterns displayed sharp peaks at 2ɵ = 31.7°, 35.5, 37, 39, 45.6, 55.3, 62.8°, 68°, and 69.31°, which can be indexed to (100), (002), (101), (102), (110), (103), (200), (112), and (201), respectively [28]. It is clear that the synthesis was successful because the ZnO/C nanocomposite shared distinct peaks that are characteristic of ZnO/C.

More impressively, no impurity peaks were found in Fig. 4, confirming the effective synthesis of the nanomaterials. Because of the low-temperature processing, the nitrogen-doped porous carbon in N doped ZnO/C-600 exhibits a low degree of graphitization, according to the associated XRD pattern. The Zn element in ZIF-8 should be converted to ZnO after calcination at 600 °C, as previously reported and supported by the EDS results. Using the Debye–Scherrer formula, the average [29] grain size (D) of the produced ZnO/C nanomaterials was determined to be 27.07 nm.

**Fig. 5:**
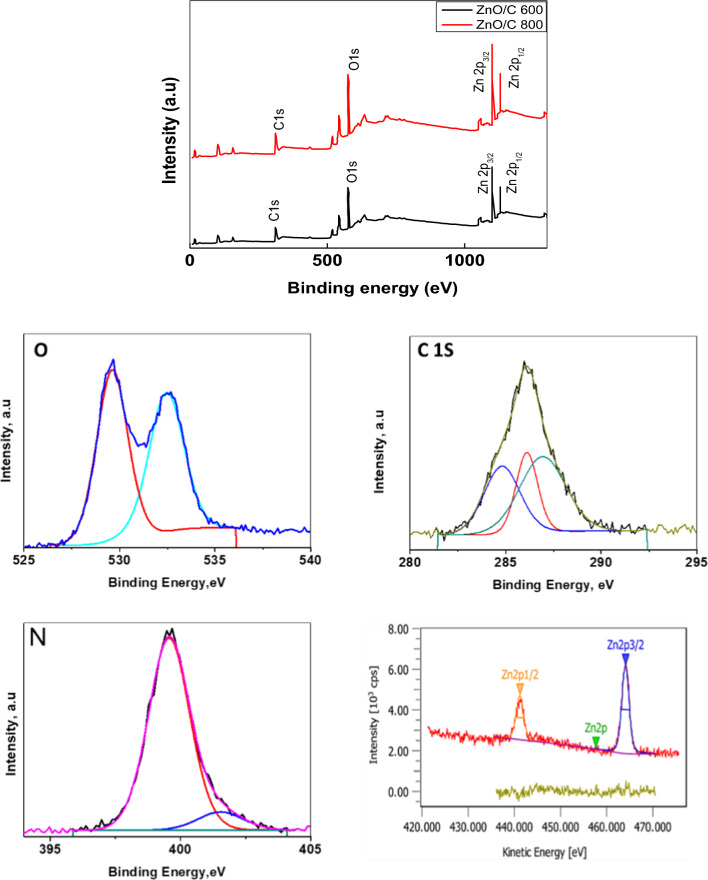
XPS spectra of N doped ZnO/C samples carbonized at different temperatures

The results of an XPS analysis have been utilized to further investigate the chemical and elemental states of N doped ZnO/C-600 and N doped ZnO/C-800. Figure 5 clearly shows that following elements C, N, O, as well as Zn—are present in the synthesized samples, which agrees with the EDS results (Fig. 2). From Fig. 5, it was possible to deconvolute the C 1s spectra of N doped ZnO/C into three main characteristic peaks at 284.7, 285.3, and 288.1 eV, which is corresponding to C sp3-C sp3, C sp2-C sp2, and C=N, respectively. Similar to this, the N 1s spectra of N doped ZnO/C can be mainly deconvoluted into three characteristic peaks located at 399.1 eV which represents the pyridinic-N, pyrrolic-N, and graphitic-N [30]. These could add to the benefits of N doped ZnO/C. In the O 1s spectra of N doped ZnO/C, there are two peaks centered at around 530.8 and 531.7 eV, which are related to the O^2−^ ions and oxygen vacancies on the surface of materials, respectively [31]. Two peaks at approximately 1021.6 and 1044.7 eV corresponding to Zn 2p_3/2_ and 2p_1/2_, are presented in the Zn 2p spectra of N doped ZnO/C. Based on the separation (23.1 eV) between the two peaks, this implies that Zn^2+^ ions can be found in N doped ZnO/C [29].

**Fig. 6:**
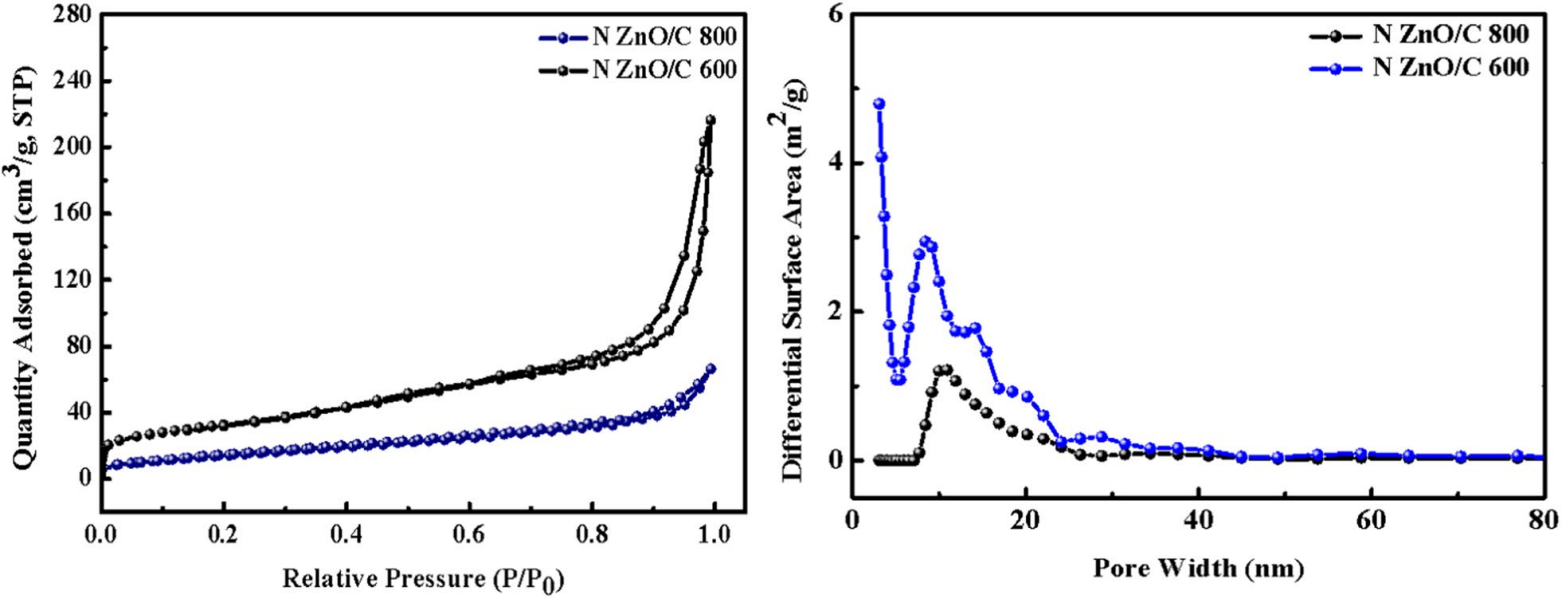
N_2_ adsorption–desorption isotherms and pore size distribution of as-synthesized materials

The N_2_ adsorption–desorption isotherms of the N doped ZnO/C-600 and N doped ZnO/C-800 were examined in order to further investigating the porosity and particular surface areas, and they are displayed in Fig. 6. As a result, a surface area analyzer was used to examine the electrodes porosity. The type IV isotherm showed a typical H3 hysteresis loop in the P/Po range of 0.7–1.0 confirming the presence of mesoporous and microporous structures as result of activation post-treatment [32]. The presence of micropores as well as mesopores in the samples may be related to the disintegration of Zn-MOF's organic framework and the release of volatile gases including carbon dioxide and water. N doped ZnO/C-600 had the highest specific surface area (S_BET_) of 609.2 m^2^/g. This might be a consequence of the ZIF8 framework disintegrating and polyhedral ZnO forming over 600 °C. Additionally, as the calcination temperature increased, the rate of framework collapse accelerated, further reducing the specific surface area of N doped ZnO/C-800. Additionally, the Fig. 6. Barrett–Joyner–Halenda (BJH) pore size distribution curve shows that the majority of the pores in N doped ZnO/C-800 and N doped ZnO/C-600 are micropores (less than 2 nm) and mesopores (2–8 nm). Mesopores typically have pores that are 3.0 nm in size, which is advantageous for fast molecular mass transfer and ion diffusion. N doped ZnO/C-600 and N doped ZnO/C-800 are anticipated to have adsorption capabilities because of their high specific surface area and the synergistic effects of many pores of varied sizes.

**Fig. 7:**
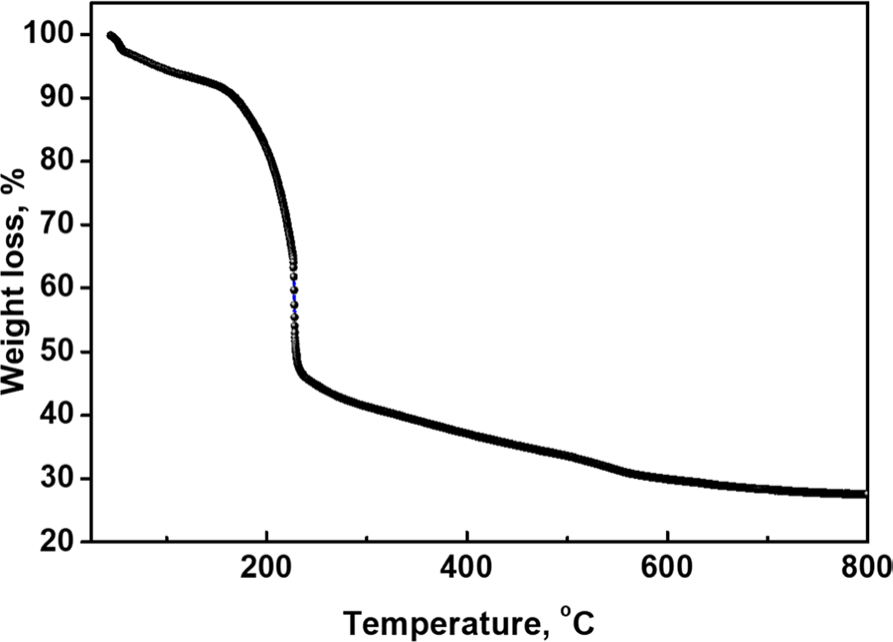
Thermogravimetric analysis (TGA) for ZIF-8

Figure 7 depicts the ZIF 8-derived materials temperature profile before pyrolysis process. Zif-8 has been decomposed to three stages of weight loss. The evaporation of solvent molecules accommodated in the cavities of ZIF-8 caused a weight loss of 14.18% at temperatures ranging from 0 to 200 °C. The removal of hydrogen components from the terephthalic ligand at 178–285 °C may be the cause of the 8.39% weight loss. 14.65% weight loss at 285–420 °C indicates the breakdown of oxygen components in the terephthalic ligand [33]. The degradation of the carbon component of Zif-8 was indicated by a rapid drop in weight loss of 38.02%. According to TGA findings, Zif-8 has been largely disintegrated after being heated to 600° C.

### Adsorption isotherm studies

**Fig. 8:**
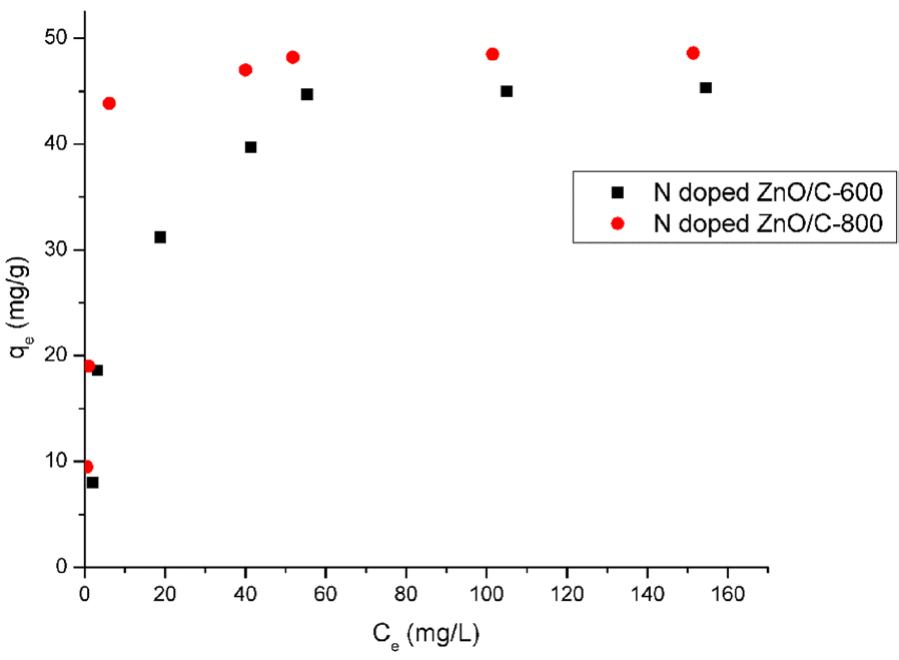
Adsorption isotherm for RBBR dye on as-synthesized samples

The adsorption isotherms were probed using a weight 10 mg of N doped ZnO/C samples for RBBR dye at concentrations (20–200) mg/L and set at temperature 308 K. The equilibrium concentration (C_e_) was outlined against the adsorption capacity at equilibrium (q_e_) to procure the general shape of the adsorption curves [34, 35] as displayed in Fig. 8 which demonstrates that the adsorption efficiency increased with increasing RBBR dye concentration, then settled saturation at high concentrations. The equilibrium adsorption results suggested that active N doped ZnO/C-800 has a somewhat better adsorption capacity of 49.3 mg/g. This arised as a result of the increment in the driving force of the concentration gradient and its high surface area as mentioned previously. If the dye concentration in solution is bigger, the active sites of adsorbent would be contacted by more dye ions and the process of sorption would be properly carried out. Hence, the mechanism of RBBR dye uptake related mainly to the large surface area, meso- and microporosity of prepared samples which enhanced after activation approach. Additionally, the dispersive interactions between the delocalized π-electrons on the surface of activated samples acquired from the surface functionality and the free electrons of the anionic dye molecules play a dominant role in the adsorption mechanism. Langmuir and Freundlich are two of the most well-known isotherms for modeling dye diffusion among liquid and solid phases [34].

#### Langmuir adsorption isotherm study

**Fig. 9:**
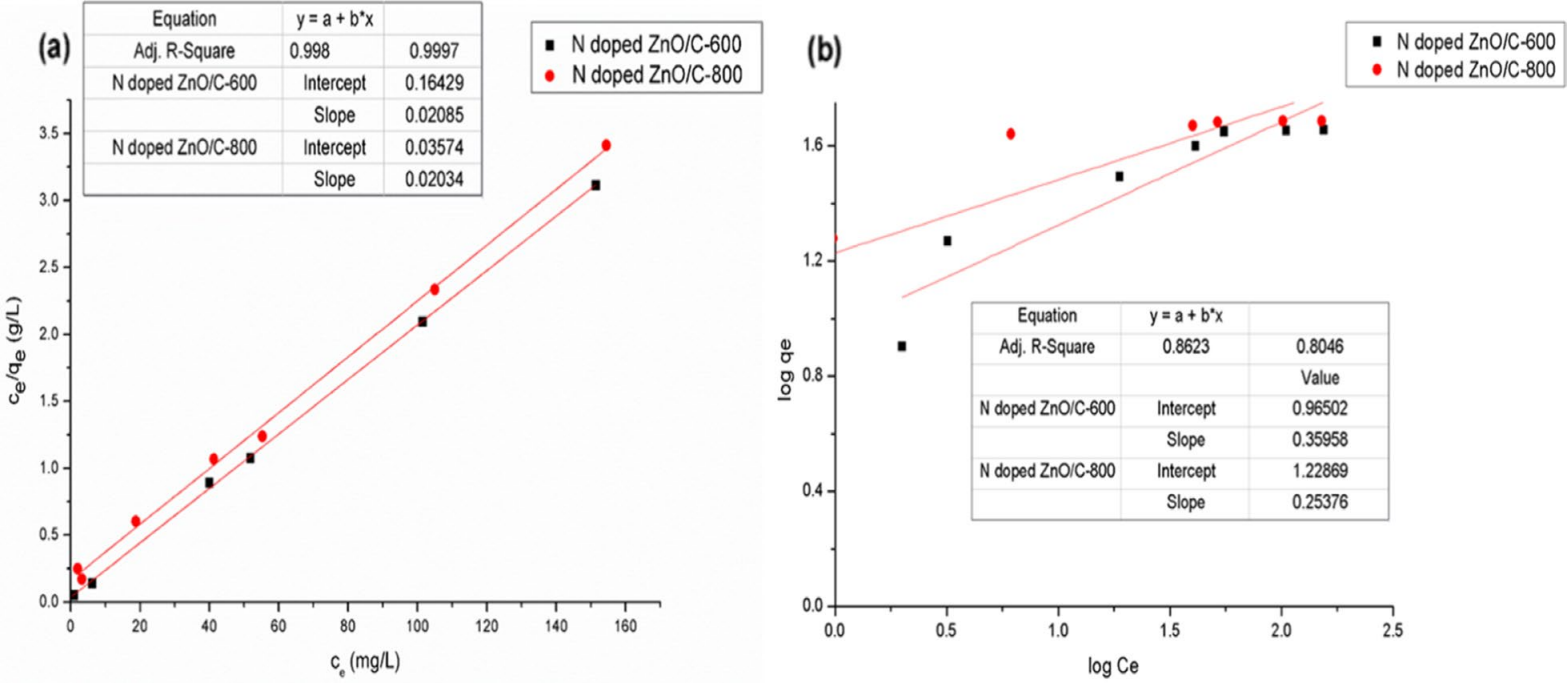
**a** Langmuir and **b** Freundlich adsorption isotherm patterns for RBBR dye on as-synthesized N doped ZnO/C nanoparticles

Langmuir isotherm model for monolayer adsorption is assumed that once an adsorbate has filled a site, there is no additional adsorption at that site resulting in a distinctive plateau in the curve, and there is no transverse contact and steric hindrance between adsorbed species [36, 37]. The model can be represented as follows:

2 $$\frac{{{\text{C}}_{{\text{e}}} }}{{{\text{q}}_{{\text{e}}} }} = \frac{1}{{{\text{K}}_{{\text{L}}} {\text{q}}_{{\text{m}}} }} + \frac{{{\text{C}}_{{\text{e}}} }}{{{\text{q}}_{{\text{m}}} }}$$

where q_m_ is the dye maximal adsorption capacity per gram of adsorbent (mg/g), and K_L_ is the Langmuir constant (L/mg), which is sensitive upon the affinity of binding sites. C_e_/q_e_ was figured against C_e_ to demonstrate the experimental data (Fig. 9a). The intercept and slope values of the plot were employed to compute Langmuir constant (K_L_), and maximal adsorption capacity per unit of the adsorbent (q_m_) [38]. The dimensionless equilibrium parameter (separation factor, R_L_) was exploited as an adsorption experimental indicator through further evaluation of Langmuir equation, in which ${\mathrm{C}}_{o}$ is the initial concentration of maximum removal efficiency [39].

3 $${\text{R}}_{{\text{L}}} { } = \frac{1}{{1{ } + {\text{ K}}_{{\text{L}}} {\text{C}}_{o} }}$$

The adsorption characteristics of the dye with the adsorbent is examined by R_L_ value. The adsorption process is unfavorable if the R_L_ value is larger than 1, however R_L_ = 0 implies that adsorption process is irreversible. Whether the 0 < R_L_ ≤ 1 value suggests a uniform adsorption [39, 40]. The R_L_ values for RBBR dye sorption on the assessed N doped ZnO/C-(600 and 800) were 0.13 and 0.03, respectively in the present research verifying that N doped ZnO/C samples are suitable for RBBR dye adsorption underneath the simulated conditions. The Langmuir variables were presented in Table 2 along with the correlation coefficient (R^2^). All the adsorption isotherm patterns of RBBR dye were expected to be adapted with standard Langmuir in shape according to R^2^ values which show the strong linear relationship (0.998 and 0.9997 close to 1). The fitting of the experimental data to the Langmuir isotherm pattern reveals that the tested materials were homogeneous and dye molecules can form coverage on the outer surface.

**Table 2:** Summary of adsorption isotherm constants for the reactivity of RBBR dye onto prepared N doped ZnO/C

Adsorbents	Langmuir				Freundlich		
	K_L_ (L/mg)	Q_m_ (mg/g)	R_L_ (mg/L)	R^2^	K_F_ (mg/g)	n	R^2^
N doped ZnO/C-600	0.13	48.1	0.13	0.998	9.23	2.8	0.8623
N doped ZnO/C-800	0.57	49.3	0.03	0.9997	16.9	3.9	0.8046

#### Freundlich adsorption isotherm study

The Freundlich isotherm is an empirical model in which adsorbed molecules interact on heterogeneous surfaces with homogeneous energy distribution (multilayer adsorption) [41, 42]. Also according to this pattern, adsorbate concentration on adsorbent raises as long as adsorbate concentration in the solution increases without reaching saturation [43]. Freundlich isotherm equation is represented as follows.

4 $${\text{logq}}_{{\text{e}}} { } = \log {\text{K}}_{{\text{F}}} + \frac{1}{{\text{n}}}{\text{logC}}_{{\text{e}}}$$

To ascertain the Freundlich isotherm, the experimental measurements of log q_e_ were figured against log C_e_ (Fig. 9b). K_F_ (mg/g) and n are the Freundlich isotherm constants related to adsorption intensity calculated from the intercept and the slope value of the plot. The adsorption process is outstanding when the 1/n value is about 0.1 and less than 0.5. If the value is among 0.5 and 1, the process is straightforward to adsorb and if the value is greater than 1, it is challenging to [44]. In this research, the 1/n value for N doped ZnO/C-600 and 800 is (0.35 and 0.25), adequately. In Table 2, the Langmuir and Freundlich parameters were evaluated and compiled. Hence, these results mean that RBBR dye adsorption is in a stronger correlation with the Langmuir isotherm than Freundlich model. The results of this study are comparable with that of prior studies as summarized in Table 3. According to their findings, N doped ZnO/C is a feasible low-cost adsorbent to eliminate dyes from aqueous solution.

**Table 3:** Comparative study of maximum adsorption capacity among our prepared specimens and other adsorbents

Adsorbent	Modification	Q_m_ (mg/g)	References
N doped ZnO/C-600	Chemical-Activation	48	This work
N doped ZnO/C-800	Chemical-Activation	49.3	This work
Zinc oxide powder	Calcination	38.9	[45]
Bottom Ash	Untreated	34.60	[27]
Bottom Ash	H_2_O_2_-treated	30.86	[27]
Bottom Ash	Physical-activated	43.29	[27]
OBBL/PET/24.5%/P-mTol	–	8.9	[46]
Green algae active	–	68	[47]
Green algae inactive	–	95.2	[47]
Wheat brane	–	97.1	[48]
Wheat brane	–	6.4	[49]

### Adsorption dynamic study

**Fig. 10:**
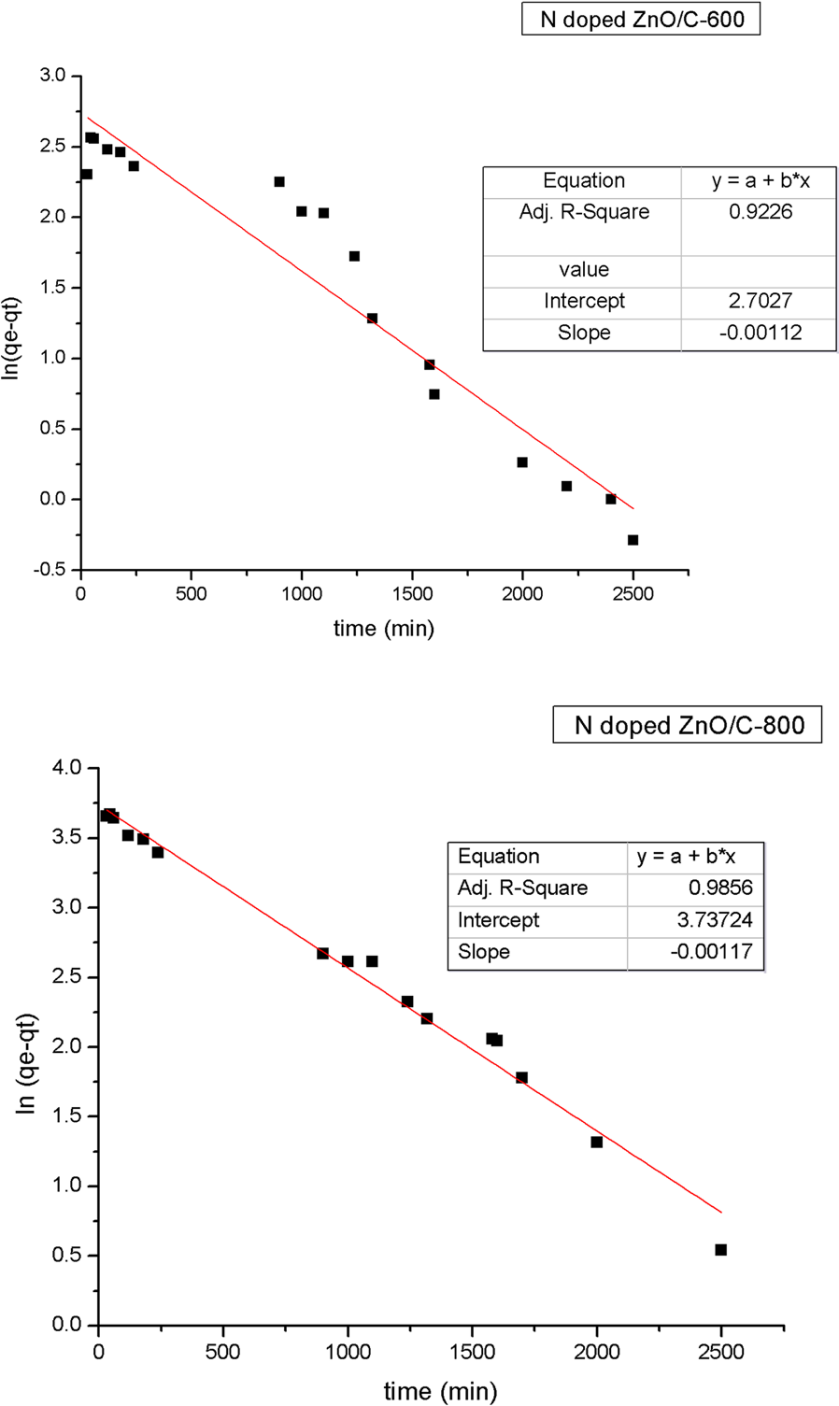
Lagergren pseudo-first order equation of RBBR dye

The impact of contact time on the adsorption of RBBR dye at constant concentration (50 mg/L) was investigated with the findings given in Fig. 10. In the beginning stages, the proportion of removed dyes increased substantially. However as the contact time increased, the percentage of detached dyes steadily climbed until equilibrium. The optimum time to reach equilibrium was 48 h. The data gained from adsorption kinetic experiments were utilized in simulations of pseudo-first-order, pseudo-second-order, and intra-particle diffusion models to assess the sorption process of dye onto N doped ZnO/C samples.

#### The first-order kinetic model

The adsorption rate constant was calculated from the first-order kinetic term given by [50]:

5 $$\ln \left( {{\text{q}}_{{\text{e}}} - {\text{q}}} \right) = \ln {\text{q}}_{{\text{e}}} {-}{\text{K}}_{1} {\text{t}}$$

where q_e_ and q are the adsorption capacity of dye adsorbed (mg/g) at equilibrium and at time t (min) adequately, and k_1_ is the adsorption rate constant (min^−1^). The computed correlation coefficient (R^2^) for the first-order kinetic model is closer to unity as shown through Fig. 10, in addition to the experimental and theoretical adsorption capacity at equilibrium seemed to be the same whilst using N doped ZnO/C-800. As a consequence, the adsorption kinetics for RBBR dye could be better modeled by first order kinetics for N doped ZnO/C-800.

#### The second-order kinetic model

**Fig. 11:**
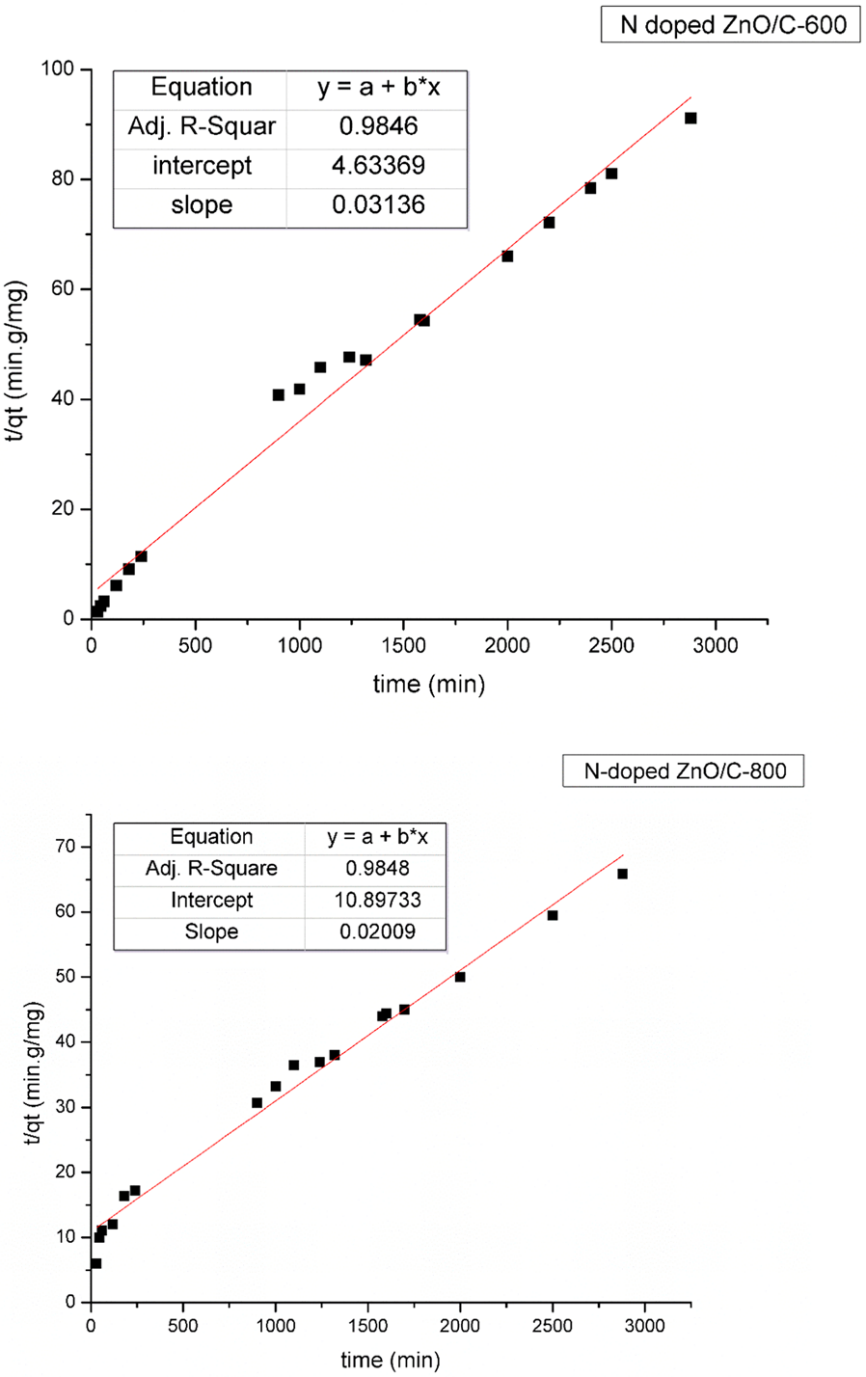
Lagergren pseudo-second order equation of RBBR dye

The second-order kinetic model is termed as [51]:

6 $$\frac{{\text{t}}}{{\text{q}}} = \frac{1}{{{\text{K}}_{2} {\text{q}}_{{\text{e}}}^{2} }} + \frac{{\text{t}}}{{{\text{q}}_{{\text{e}}} }}$$

where k_2_ (g/mg min) (the second-order constant) may be derived empirically from the intercept of plotting (t/q) against time (t) (Fig. 11). The value of q_e_ derived from the second order equation is an approximation to its practical value according to N doped ZnO/C-600. Therefore, the adsorption kinetics for RBBR dye might be better simulated by second order kinetic model with N doped ZnO/C-600. Table 4 comprises the practically and theoretically estimated q_e_ as well as k1, and R^2^.

**Table 4:** Kinetic constants for RBBR dye removal by N doped ZnO/C samples

Adsorbent	N doped ZnO/C-600			N doped ZnO/C-800		
**Temperature (K)**	308			308		
**q**_**e**_ **(exp) (mg/g)**	31.6			43.7		
**Pseudo first-order**	**q**_**e**_ **(mg/g)**	**R**^**2**^	**K**_**1**_ **(min**^**−1**^**)**	**q**_**e**_ **(mg/g)**	**R**^**2**^	**K**_**1**_ **(min**^**−1**^**)**
	14.9	0.9226	1.1 × 10^–3^	43.5	0.9856	1.2 × 10^–3^
**Pseudo second-order**	**q**_**e**_ **(mg/g)**	**R**^**2**^	**K**_**2**_** (g/mg.min)**	q_e_ **(mg/g)**	R^2^	**K**_**2**_** (g/mg.min)**
	31.8	0.9846	2 × 10^–4^	49.7	0.9848	3.7 × 10^–5^
**Intra-particle diffusion constants**	**K**_**id**_ **(mg/g.min**^**0.5**^**)**	**R**^**2**^	**C**	**K**_**id**_ **(mg/g.min**^**0.5**^**)**	**R**^**2**^	**C**
	0.29	0.989	16.4	0.89	0.9904	0.006

#### Intra-particle diffusion study

Intra-particle diffusion process is frequently the rate determine step in many adsorption processes. It is most likely show how the adsorbate species are carried from the bulk of the solution into the solid phase. The potential of intra-particle diffusion process was addressed through using the Weber–Morris plot [52] who elucidated that in most adsorption processes, the uptake fluctuates exponentially with t^1/2^ rather than with the contact time (t) according to the following equation:

7 $${\text{q}}_{{\text{t}}} = {\text{K}}_{{{\text{id}}}} {\text{ t}}^{\frac{1}{2}} { } + {\text{ C}}$$

where k_id_ is the intra-particle diffusion rate constant (mg/g min^0.5^) determined from the slope of a graph of (q_t_) versus (t^0.5^), and C is a constant computed from the intercept when the adsorption mechanism follows the intra-particle diffusion process.

**Fig. 12:**
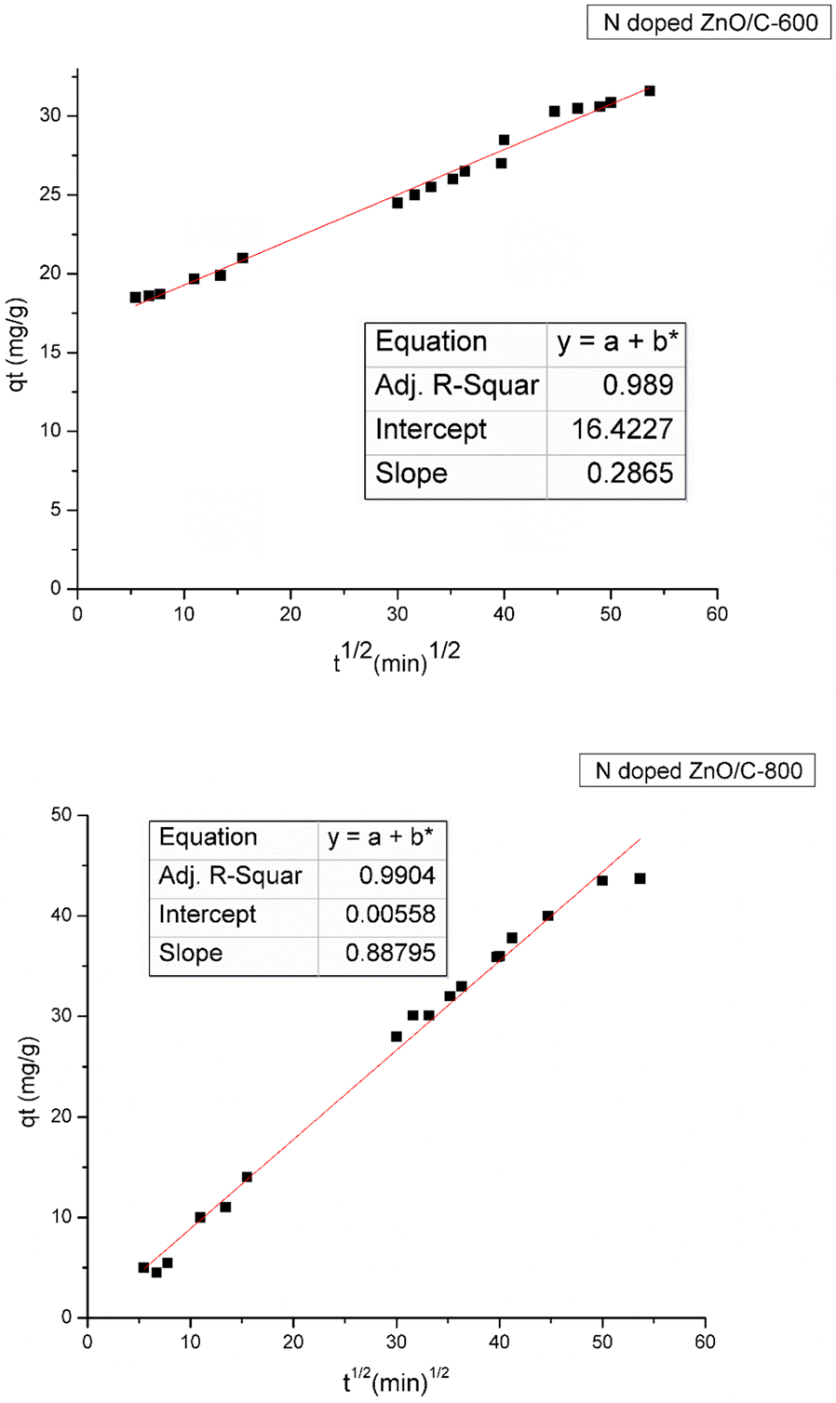
Weber and Morris intra-particle diffusion plots for removal of RBBR dye

The findings of q_t_ were linearly correlated with the values of t^1/2^ as seen from Fig. 12. The linear plots were ascribed to the large pore diffusion which was the accessible site for adsorption. This was attributable to the instantaneous adsorption on the most readily accessible adsorbing sites on the adsorbent surface. Table 4 lists the values of k_id_ and C as captured from the slope and the intercept of the straight line. The R^2^ values are close to unity recommending that the intra-particle diffusion mechanism was being used. The intercept values (Table 4) denoted an idea about the boundary layer thickness, with a larger intercept for N doped ZnO/C-600 (16.4 mg/g min^0.5^) and linear plot indicating a greater boundary layer impact [53]. However, the intercept value of N doped ZnO/C-800 plot was close to zero representing that pore diffusion is not the step that control the overall rate of mass transfer at start of batch adsorption for this sample, in the early stages of adsorption, film-diffusion control may have occurred and ceased [54].

### Adsorption thermodynamic study

**Fig. 13:**
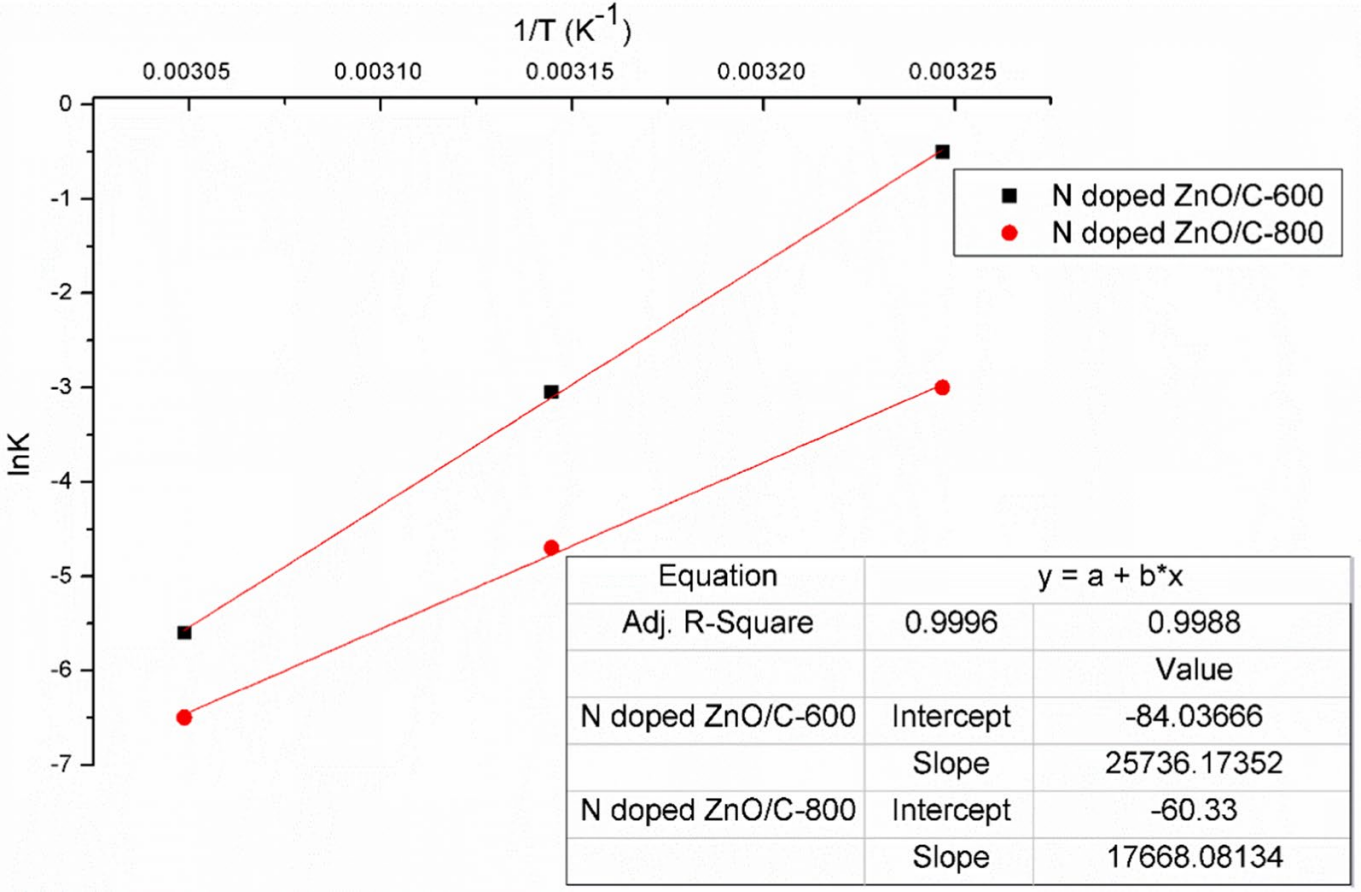
Vant Hoff equation for adsorption performance of RBBR dye on prepared N doped ZnO/C

Thermodynamic parameters including standard enthalpy (Δ*H*°), standard Gibbs free energy (Δ*G*°) and standard entropy (Δ*S*°) disclose details on the intrinsic energetic changes involved with adsorption [55]. The values of Δ*H*° and Δ*S*° are acquired by Vant Hoff equation:

8 $${\text{ln K}}_{{\text{L}}} = { }\frac{{{\Delta S}^\circ }}{{\text{R}}} - \frac{{{\Delta H}^\circ }}{{{\text{RT}}}}$$

where *R* (8.314 J/mol K) is the universal gas constant, *T* (K) is the absolute solution temperature, and *K*_*L*_ (L/mg) is the Langmuir isotherm constant. The Δ*H*° and Δ*S*° can be evaluated from the gradient and intercept of (ln*K*_*L*_) versus (1/*T*) from Fig. 13.

Equation (9) may be used to compute Δ*G*° value.

9 $$\Delta {\text{G}}^\circ = { }\Delta {\text{H}}^\circ - {\text{T}} \cdot \Delta {\text{S}}^\circ$$

**Fig. 14:**
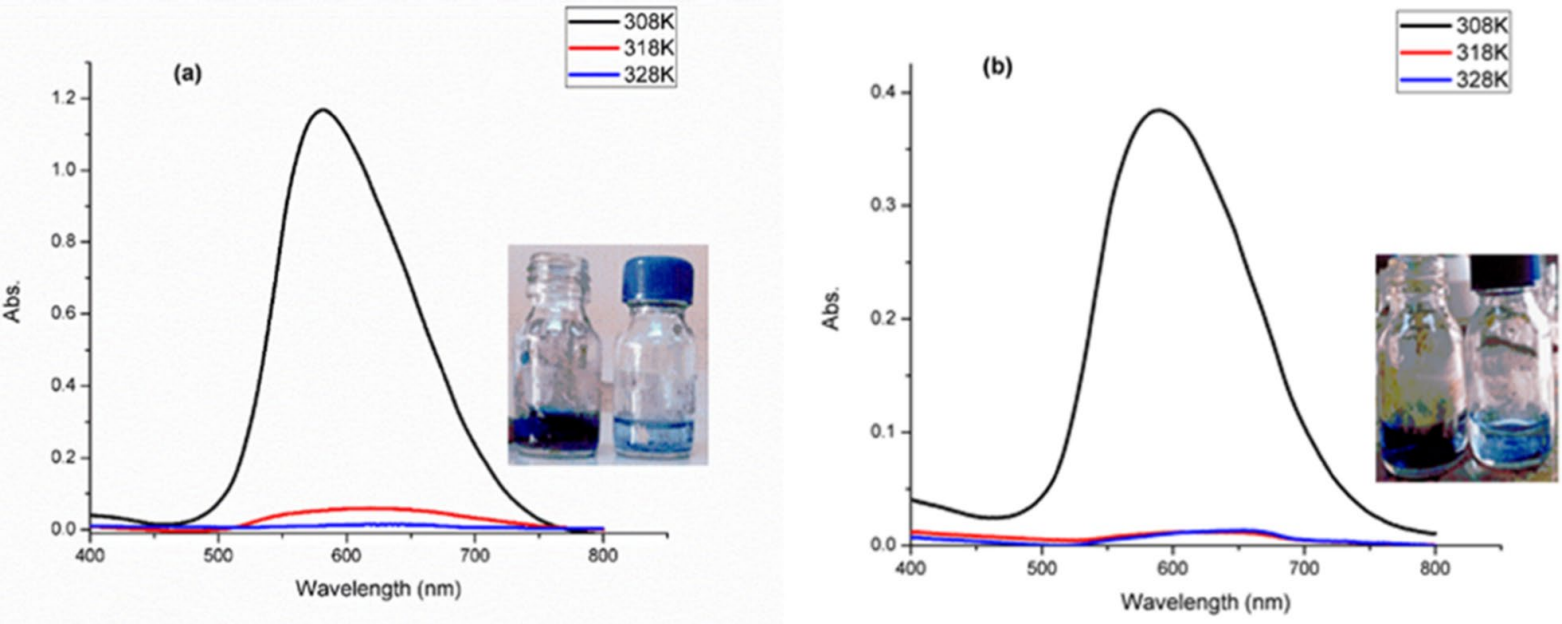
Absorbance curves representing the impact of solution temperature on RBBR dye adsorption (50 mg/L) by **a** N doped ZnO/C-600, **b** N doped ZnO/C-800 with photo-pictures

As illustrated from Fig. 13, the increment of the solution temperature from 308 to 328 K boosted the elimination of dye over N doped ZnO/C specimens. The non-spontaneous adsorption characteristics have been supported by the positive values of ΔG° as shown from Table 5. The ΔG° values raised as the temperature raised suggesting stronger driving force and therefore increased adsorption capacity at higher temperatures. Increasing the temperature enhanced the dye molecule diffusion all over the exterior and interior boundary layer of adsorbent because of the reduction in solution apparent viscosity [54, 56]. Furthermore at higher temperature, more dye molecules had sufficient energy to interact with adsorbent active sites, the dye mobility increased and the dye molecules were allowed to permeate the pores of adsorbent [55]. The negative values of ΔH° supported the exothermic nature, while (Δ*S*°) values pointed out the bonding process of particles adsorbed on the surface of N doped ZnO/C with a negative value exhibiting less randomness and a more regular pattern for RBBR dye adsorption at the solid/solution interface [57]. Figure 14 depicts the fluctuation of adsorption capacity at different temperatures with the same behavior for both N doped ZnO/C-(600 and 800).

**Table 5:** Thermodynamic parameters for the adsorption of RBBR dye on prepared samples

Adsorbent	ΔH° (KJ/mole)	ΔS° (KJ/mole K)	ΔG° (KJ/mole)		
			308 K	318 K	328 K
N doped ZnO/C-600	− 214	− 0.7	+ 1	+ 8	+ 15
N doped ZnO/C-800	− 147	− 0.5	+ 7.6	+ 12.6	+ 17.6

## Conclusion

Nitrogen-doped ZnO/C-(600 and 800) samples were synthesized by directly pyrolysis in (N_2_) gas and at high temperatures of prepared (ZIF-8), followed by chemical activation. According to the findings of surface characteristics, the as-synthesized N doped ZnO/C featured a rhombic dodecahedron morphology with a uniform particle size of about 100 nm, the chemical activation post-treatment of N doped ZnO/C enriched the porosity of produced samples and greatly enhanced the surface area (609.2 m^2^/g). Porosity and surface area greatly effect on adsorption capacity. This work clearly demonstrates the potential of combining nitrogen-doped ZnO/C as a core material that can be used for environmental remediation. Langmuir model was followed by the equilibrium data. The kinetic studies showed that RBBR dye well fitted to pseudo first order kinetic model for N doped ZnO/C-800, and pseudo second order kinetic approach for N doped ZnO/C-600. The non-spontaneous and exothermic nature of the RBBR dye adsorption onto prepared samples was confirmed by the thermodynamic parameters of Δ*G*° and Δ*H*°, positive and negative values, adequately. The adsorption capacity for RBBR dye (49.3 mg/g) is an increasing function of the extent of surface functionalization that influenced the dispersive interactions between the delocalized π–electrons on the surface of prepared samples and the free electrons of the anionic dye molecules. Additionally, the microporosity, mesoporosity and the high surface area which acquired from surface activation are the factors defining the adsorption capacity for RBBR in this study.

## Data Availability

All data and materials are available.
